# Association of asthma with coronary heart disease: A meta analysis of 11 trials

**DOI:** 10.1371/journal.pone.0179335

**Published:** 2017-06-13

**Authors:** Lida Wang, Shuyan Gao, Mingdong Yu, Zhixin Sheng, Wei Tan

**Affiliations:** 1 Department of E.N.T, Weifang People’s Hospital, Weifang, China; 2 Department of Hematology, Weifang People’s Hospital, Weifang, China; 3 Department of Orthopaedics, Weifang People’s Hospital, Weifang, China; 4 Department of Respiration, Weifang People’s Hospital, Weifang, China; South Texas Veterans Health Care System, UNITED STATES

## Abstract

**Purpose:**

While the relationship of asthma and coronary heart disease (CHD) (a specific manifestation of cardiovascular disease) has not been described consistently, we tried to defined this relation and explore the influence of gender and asthma status (child- and adult-onset asthma) on this issue.

**Methods:**

We searched published reports that described the relationship of asthma and CHD.

**Results:**

Eleven trials were identified, covering 666,355 subjects. Asthma overall was significantly associated with CHD both for prospective trials (HR 1.34 [1.09,1.64], P = 0.005) and for retrospective trials(OR 1.29 [1.13,1.46], P = 0.001), when compared to individuals without asthma. Subgroup analysis split by gender indicated that females with asthma were significantly associated with CHD (HR 1.40 [1.20,1.62], P<0.001), but males with asthma were not significantly related with CHD (HR 1.19 [0.98,1.44], P = 0.07). For the four subgroups (Females with adult-onset asthma,males with adult-onset asthma,females with child-onset asthma,and males with child-onset asthma), pooled analysis of two trials indicated that only females with adult-onset asthma were significantly associated with CHD (HR 2.06 [1.32,3.19], P<0.001).

**Conclusions:**

Our data indicated that asthma was associated with CHD, and the relationship between them seemed to derived mostly from females with adult-onset asthma. Considering the limits of our study, these findings should be taken with caution.

## Introduction

Asthma characterized by bronchospasms and airflow obstruction is a chronic inflammatory disease of the airway and affects about 300 million people worldwide. Inflammation has been reported to play an important role in the pathogenesis of atherosclerosis [1–3]. Although there had been many trials examining the relationship of impaired lung function and cardiovascular disease (CVD)[4–9], only few trials relating asthma to CVD were reported. Recently, several trials supporting the relationship of asthma and CVD has been reported[10–11]. All the papers supported the relationship of asthma and CVD, but they all related distinct specific CVD outcomes (myocardial infarction, stroke, coronary heart disease (CHD), hypertension, heart failure or angina) to asthma, sometimes with distinct results for the same endpoint. The study by Lee suggested that CHD is the principal manifestation of CVD related with asthma[12], and supported by some trials [13–17]. Because of the chronic inflammatory nature of both asthma and CHD, inflammatory reaction might be a potential link between the CHD and asthma. Meanwhile, asthma was not significantly associated with CHD in some other trials[18–22]. So, we undertook this pooled analysis to clarify this relationship of asthma and CHD. There were some trials that presented the possible association split by gender, indicating this association may be stronger for females[13,15,17]. So, we also undertook subgroup analysis to explore the influence of gender on this association. Furthermore, asthma is not one unique disease, but a aggregation of different underlying subtypes with distinct causes [23–25]. Adult- and child-onset asthma vary regarding gender distribution, asthma triggers, and systemic inflammation[23–25]. In this pooled analysis, we also examined the possible influence of age of asthma onset on this association.

## Methods

### Literature search strategy

The Cochrane Controlled Trial Register, Embase, Medline, and the Science Citation Index were searched using the medical subject headings “asthma”, “cardiovascular disease” “coronary heart disease”, and “ischemic heart disease”. Reference lists of selected reports were also hand-searched. This pooled analysis was approved by the institutional review boards of Weifang People’s Hospital, in accordance with the Helsinki Declaration.

### Selection of studies

Trials were included for this analysis if they met the following criteria: (1) They were published up to October, 2016. (2) They tried to clarify the association of asthma and coronary heart disease. (3) They had to provide the data of hazard ratio (HR) or odds ratio (OR) for CHD when compared asthma patients with individuals without asthma. Multiple reports about a single trial were considered as one. All potential trials were reviewed by two investigators separately(L.D.W and Z.X.S.).

### Outcome measures

The primary outcome was to clarify the association of asthma and CHD, and evaluate the possible influence of gender and age of asthma onset on this association.

### Quality assessment

The Newcastle-Ottawa Scale (S1 Table) was used to assess the quality of each enrolled trial. This measure assesses aspects of methodology in observational trials associated with study quality, including case selection, comparability of population and ascertainment of exposure to risks.

### Statistical analysis

All these analyses were undertaken using a random-effects model which could provided a more conservative result. The heterogeneity among these trials was evaluated using Cochrane χ^2^ test and quantified with the *I*^2^ statistic. We also undertook subgroup analyses to sought the source of heterogeneity. Publication bias was evaluated with Egger's test. All meta-analyses were undertaken with Review Manager (version 5.3; The Cochrane Collaboration, Oxford, England) and Stata ver. 12.0 software (College Station, TX). Statistical significance was defined as a P value of less than 0.05.

## Results

**Fig 1:**
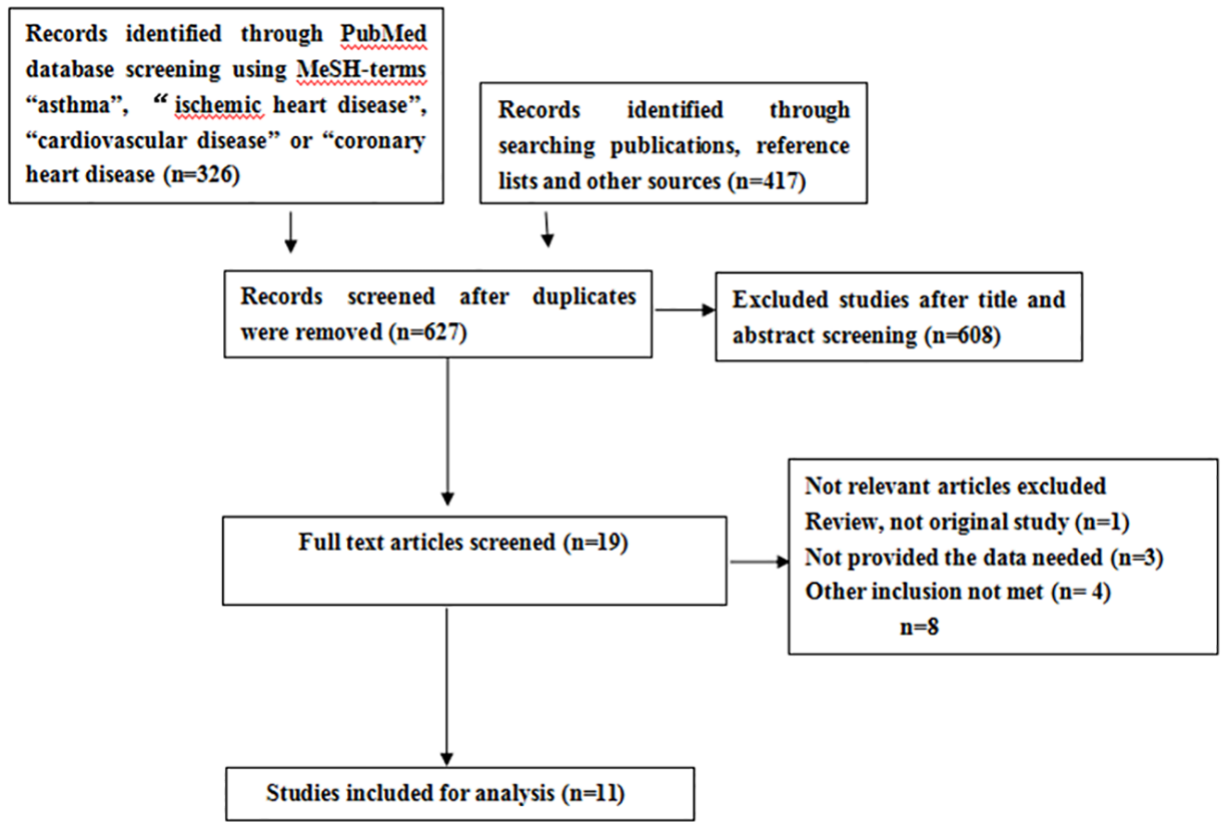
PRISMA flow diagram.

A comprehensive search of the Cochrane Controlled Trial Register, Embase, Medline, and the Science Citation Index attained 627 articles, of which 11 trials met the predefined inclusion criteria (Fig 1), covering 666,355 subjects totally[12–22]. Their characteristics were summarized in Table 1. These studies quality assessed by Newcastle-Ottawa Scale items was shown in S2 Table.

**Table 1:** Characteristics of included studies.

year	Study type	Study Region	Study population	Study years	Mean Age		No. of Subjects	Adjusted factors
					Asthma	Nonasthma		
Chung 2014[13]	Retrospective	Taiwan	Communities	1996–2011	50.5	50.9	38 840	Age, sex, and comorbidities of hypertension,Diabetes, hyperlipidemia, stroke, heart failure, COPD and smoking.
Colak 2015[14]	Prospective	Denmark	Communities	2003–2013	53/60/56*	56	40 649	Age, sex, body mass index, leisure time physical activity, education,annual household income, alcohol consumption, cumulative tobacco consumption, systolic and diastolic blood pressure, total cholesterol, low-density lipoprotein cholesterol, high-density lipoprotein cholesterol, triglycerides, use of cholesterol-lowering medication, and presence of diabetes.
Iribarren 2004[18]	Retrospective	USA	Communities	1964–1973	40 for men 41 for women	41 for men 40 for women	13047	Age, race/ethnicity, education level, smoking status, alcohol consumption, body mass index, serum total cholesterol, white blood cell count, hypertension, diabetes, parental history of coronary heart disease, and occupational exposures
Iribarren 2012[15]	Prospective	USA	Communities	1996–2008	NA	NA	407 190	Diabetes, hypertension, hyperlipidemia, body mass index, and smoking status
Lee 2012[12]	Retrospective	USA	Communities	1999–2006	53.1/37.7^▲^	49.9	16943	Age, systolic blood pressure, HDL cholesterol, BMI, hs-CRP, smoking, and diabetes mellitus.
Liss 2000[19]	Retrospective	Canada	Hospitalization	1980–1996	NA	NA	2400	Period of birth, time period of accident, and sex.
Onufrak 2008[20]	Prospective	USA	Communities	1987–2001	53.3/55.4^★^ 52.9/54.3^●^	54.3^☆^ 53.7^○^	14 567	Age,body mass index, black race,diabetes mellitus, hypertension, education level, low-and high-density lipoprotein cholesterol, and physical activity.
Prosser 2010[16]	Retrospective	British Columbia	Communities	1996/97	NA	NA	111780	Adult service user (ASU) population, the age distribution.
Schanen 2005[21]	Prospective	USA	Communities	1987–1998	54	54	13501	Age, sex, race/centre, HDL cholesterol, LDL cholesterol, systolic blood pressure, hypertension medication use, smoking status, pack years,W/H ratio, diabetes diagnosis, and sport score.
Toren 1996[22]	Prospective	Sweden	Outpatient	1962–1986	NA	NA	262	Smoking
Yun 2012[17]	Retrospective	USA	Hospitalization	1964–1983	15.1	15.1	7176	Diabetes mellitus; coronary heart disease; rheumatoid arthritis; inflammatory bowel disease.

Abbreviations: NA not available. 53/60/56* 53 for Never-Smokers asthma; 60 for Former Smokers asthma; 56 for Current Smokers asthma. 53.1/37.7^▲^ 53.1 for Adult onset asthma; 37.7 for Child onset asthma. 53.3/55.4^★^ 53.3 for men with Child-Onset Asthma; 55.4 for men with Adult-Onset Asthma. 54.3^☆^ for men with No Asthma. 52.9/54.3^●^ 52.9 for women with Child-Onset Asthma; 54.3 for women with Adult-Onset Asthma. 53.7^○^ for women with No Asthma.

**Fig 2:**
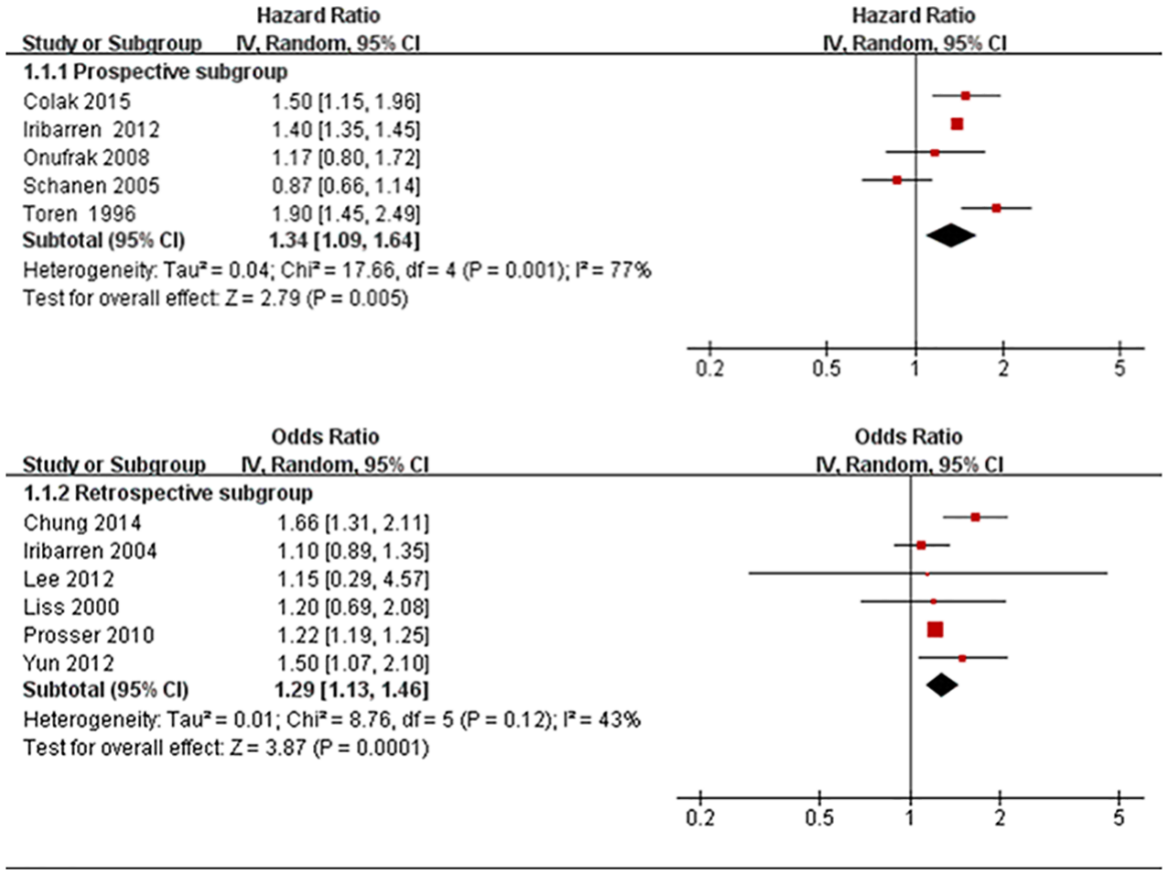
Meta-analysis of the association between asthma and coronary heart disease(CHD). HR, Hazard Ratio; OR Odds Ratio; CI, 95% confidence interval; Random, random-effects model.

As shown in Fig 2, pooled analysis indicated that asthma overall was significantly associated with CHD both for prospective trials (HR 1.34 [1.09,1.64], P = 0.005) and for retrospective trials(OR 1.29 [1.13,1.46], P = 0.001), when compared to individuals without asthma. The values for heterogeneity test were high for these prospective trials (*I*^2^ = 77%, p = 0.001), low for these retrospective trials (*I*^2^ = 43%, p = 0.12). Egger's test indicated no publication bias(P = 0.759 for prospective subgroup, P = 0.457 for retrospective subgroup). A funnel plot of publication bias was shown in S1 Fig.

**Fig 3:**
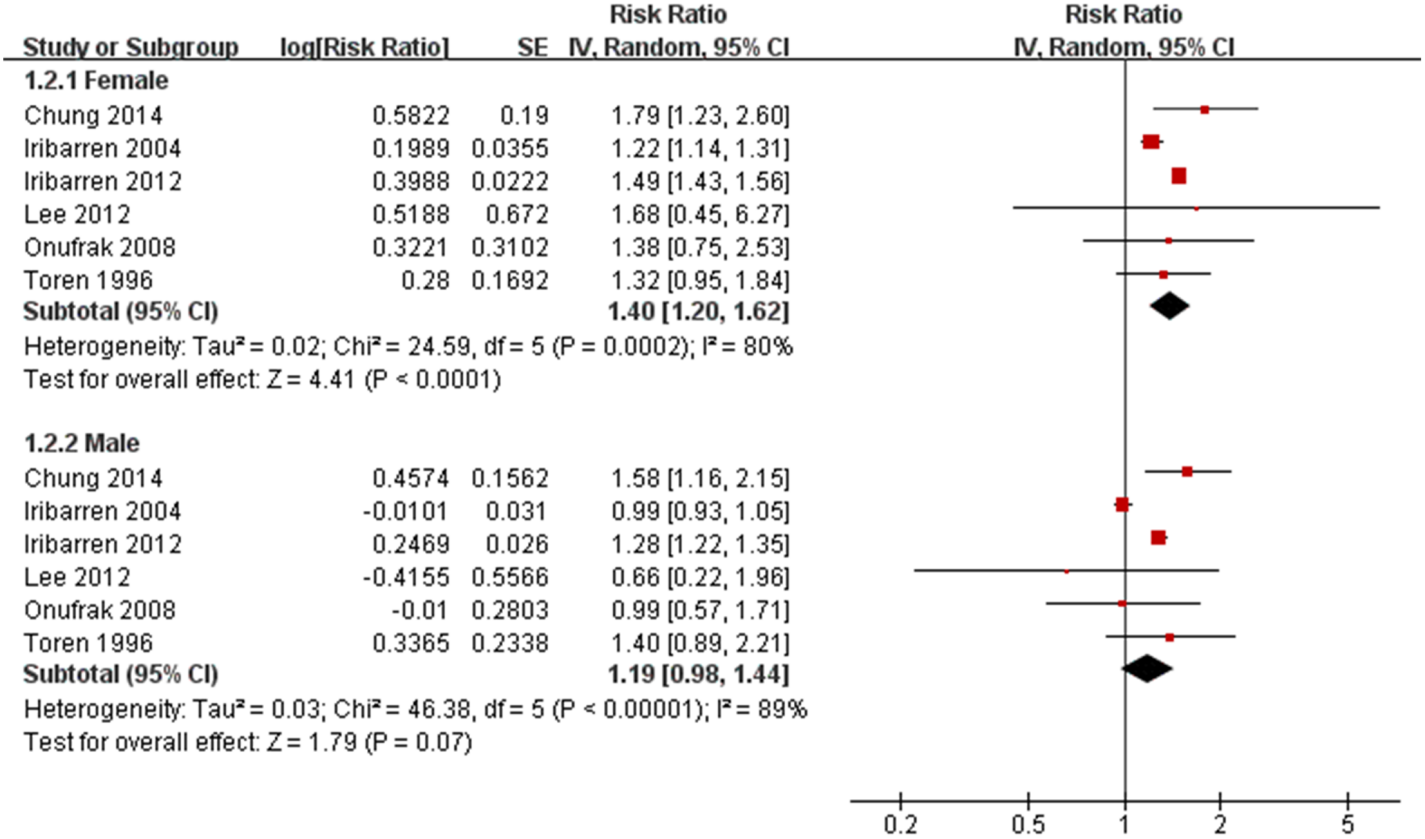
Subgroup meta-analysis of the association between asthma and coronary heart disease(CHD) by sex (females and males). HR, Hazard Ratios; CI, 95% confidence interval; Random, random-effects model.

When males with asthma were isolated,the increased risk for CHD were insignificant (HR 1.19 [0.98,1.44], P = 0.07)(Fig 3). Subgroup analysis split by gender indicated that only females with asthma attained a significant increased risk for CHD(HR 1.40 [1.20,1.62], P<0.001)(Fig 3). The significant relationship between females with asthma and CHD remained stable in sensitivity analysis when excluding any one of these trials. But only when excluding the Iribarren 2004 trial [17], the association of males with asthma and CHD became significantly(RR 1.29 [1.19, 1.40], P<0.001). The values for heterogeneity test were (*I*^2^ = 80%, p<0.001) for females with asthma subgroup,and (*I*^2^ = 89%, p<0.001) for males with asthma subgroup, respectively. No publication bias was shown for each subgroup (P = 0.90, 0.99, respectively). Meanwhile, the test for heterogeneity among genders indicated no statistical difference (P = 0.20) in Fig 3.

**Fig 4:**
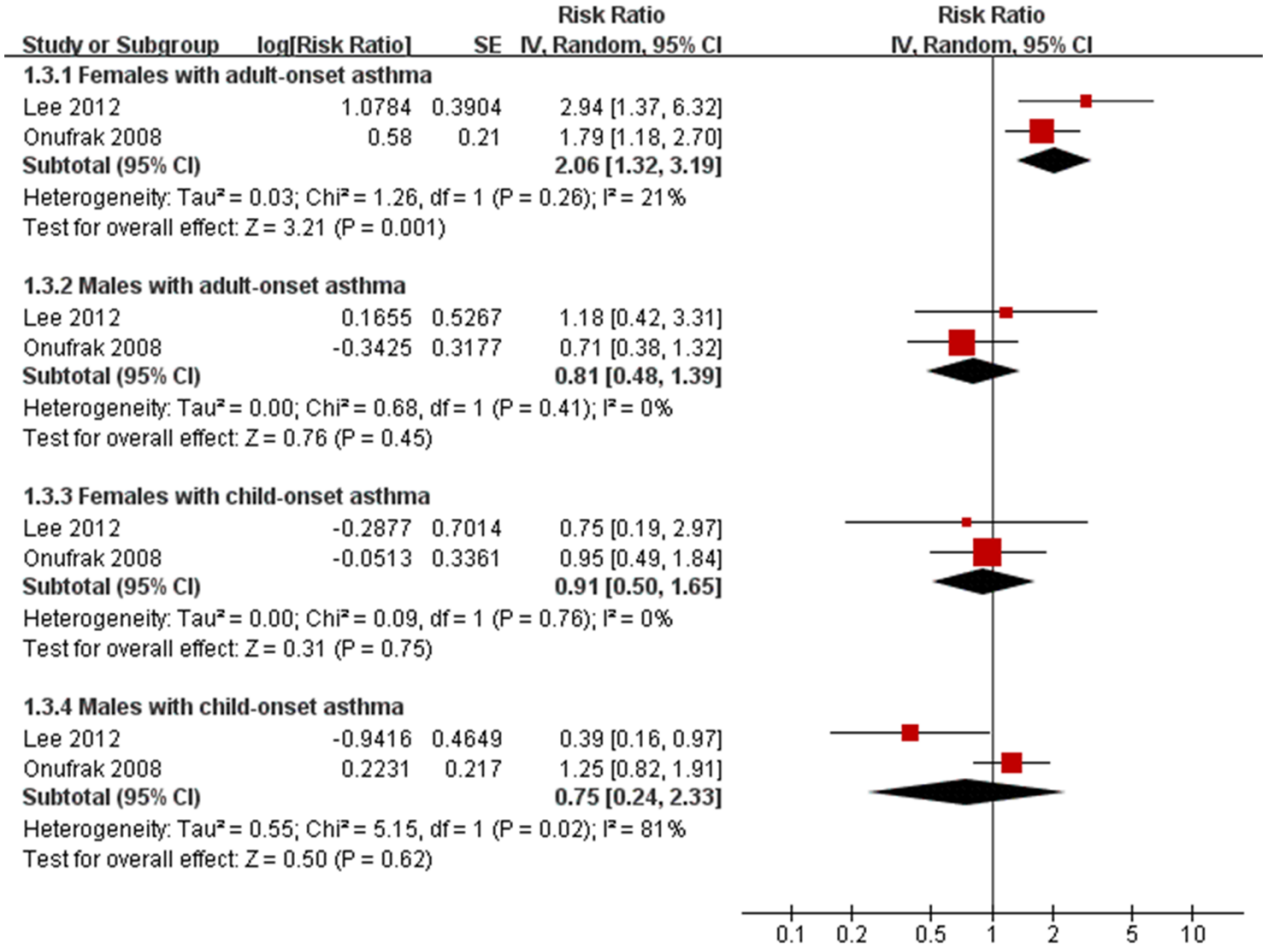
Subgroup meta-analysis of the association between asthma and coronary heart disease(CHD) by sex and age of asthma onset (child- and adult-onset). HR, Hazard Ratios; CI, 95% confidence interval; Random, random-effects model.

In gender and age of asthma onset stratified analyses, pooled analysis of two trials [12,20] indicated that a significant association with CHD was only shown in females with adult-onset asthma(HR 2.06 [1.32,3.19], P<0.001), but not in males with adult-onset asthma (HR 0.81 [0.48, 1.39], P = 0.45), females with child-onset asthma(HR 0.91 [0.50, 1.65],P = 0.75), and males with child-onset asthma(HR 0.75 [0.24, 2.33],P = 0.62)(Fig 4). All results remained stable in sensitivity analysis. The values for heterogeneity test were (*I*^2^ = 20%, p = 0.26) for females with adult-onset asthma, males with adult-onset asthma(*I*^2^ = 0%, p = 0.41),females with child-onset asthma (*I*^2^ = 0%, p = 0.76) and males with child-onset asthma(*I*^2^ = 81%, p = 0.02). In addition, there were no overall significant asthma-CHD association when pooling these two studies(HR 1.17 [0.81, 1.69], P = 0.41), and no significant association in gender-specific analysis (Males: HR 0.85 [0.51, 1.40], P = 0.10; Females: HR 1.54 [0.92, 2.57],P = 0.10).

## Discussion

Our pooled analysis indicated that patients with asthma had an increased risk of 32% for CHD occurence when compared to individuals without asthma. Since patients with asthma are usually treated with corticosteroids to control their airway inflammation, it would be proper to analyze the possible influence of steroids on this association of asthma and CHD. Since both males and females with asthma use these medicines, steroids would not totally explain why males with asthma was not found to be significantly associated with CHD. Why relationships of asthma and CHD in females are stronger than that observed in males still remains unclear. It has been suggested that estrogen enhances proinflammatory cytokines released from macrophages, monocytes, and vascular cells, all of which would intrigue asthma[26–27]. The occurrence of asthma in females was associated with shifts in estrogen levels, which incidence increased after puberty and peaked during the onset of menopause. Females with asthma may be particularly susceptible to estrogen-modulated alterations in inflammatory cytokine regulation. Because of the chronic inflammatory nature of CHD, the inflammatory cytokine alterations might be a potential link between the CHD and asthma[24]. Asthma also has been found to be associated with coronary artery spasm. Both coronary artery disease and coronary artery spasm are inflammatory diseases. Although gender is not a risk factor, more men than women have coronary artery spasm. However, racial heterogeneity shows that in Caucasians, females are more likely to develop coronary artery spasm.

Asthma has been recognized not as a uniform disease, but an aggregation of distinct conditions[23–25]. Child-onset asthma differs from adult-onset asthma in several aspects, including its distribution in males and females and its immunologic characteristics. To further elucidate possible reasons forming the relationship of asthma and CHD, we decided to split the population by two criterias: gender (males and females), age of asthma onset (adult and child-onset). Pooling analysis of two trials[12,20] had indicated that the significant association was only observed in females with adult-onset asthma, but not in females with child-onset asthma or males with either adult- or child-onset asthma. Additionally, the findings of no overall asthma-CHD association and no association in gender-specific analysis when pooling the two studies might strengthen the relationship between females with adult-onset asthma with CHD. One possible explanation for the lack of the relationship between CHD and child-onset asthma is that child-onset asthma derives from a distinct allergic basis from adult-onset asthma which has been associated with gender and environmental irritants. Because adult-onset asthma stems from more intrinsic causes inside the body, such as hormones and stiffening of chest walls. These intrinsic factors could increase CHD risk. Another possible reason was that cigarette smoking had been shown to affect adult-onset asthma, and was also a risk factor for CHD [28–30].

However, these findings should be interpreted with caution: Firstly, we used abstracted data, whereas an individual patient data-based pooled analysis would have provided a more precise estimate of the relationship of asthma and CHD. Current asthma symptoms, smoking and obesity had been shown to be important factors in phenotypes of adult-onset asthma [29–30]. They could be analyzed well with individual patient data. Secondly, medication information including dosages for asthma is lacking, because steroids could be an important confounding factor. Thirdly, the studies were relatively heterogeneous with respect to patient population, disease status, and study design. Given this clinical difference (Table 1) and statistical heterogeneity (Fig 2) among them, our decision to this association could be questioned.

The data supports the conclusion that asthma was associated with CHD, and the relationship between them seemed to derived mostly from females with adult-onset asthma. Considering the limits of our study, these findings should be taken with caution.

## Supporting information

S1 Table The Newcastle-Ottawa Scale. (DOC)

S2 Table Quality assessment of these enrolled trials (see check list in S1 Table). (DOCX)

S1 Fig Funnel plot of publication bias. (TIF)
