# Supplementary material for: Association of asthma with coronary heart disease: A meta analysis of 11 trials
Source: PLoS One. 2017 Jun 13;12(6):e0179335. doi: 10.1371/journal.pone.0179335 (PMC5469478; doi:10.1371/journal.pone.0179335)
Supplement: S1 Table — (DOC) [file pone.0179335.s001.doc]

**S1 Table. The Newcastle-Ottawa Scale**

| **Check list** |
| --- |
| ***Selection*** |
| 1. Assignment for asthema diagnosis: any criteria reported? (if yes, one star) |
| 1. Was the asthema group truly or somewhat representative of the average population with asthema disease? (if yes, one star; no star if the patients were selected or selection of group was not described) |
| 1. Was the non-asthema group drawn from the same community as the asthema group? (if yes, one star) |
| 1. Demonstration that outcome of interest was not present at the start of the study. (if yes, one star) |
| ***Comparability*** |
| 1. Group comparable for patients’s age, sex, body mass index, and presence of diabetes. (if yes, one star; no star was assigned if the two groups differed) |
| 1. Group comparable for control’s age, sex, body mass index, and presence of diabetes. (if yes, one star;no star was assigned if the two groups differed) |
| ***Outcome assessment*** |
| 1. Clearly defined outcome of interest (yes, one star for information ascertained by record linkage or interview; no star if this information was not reported or self-report) |
| 1. Was follow-up long enough for outcomes to occur? (one star if follow-up >2 years) |
| 1. Adequacy of follow-up (one star if follow-up >90%, no star if no description of those lost to follow-up) |
