# Supplementary material for: Association of asthma with coronary heart disease: A meta analysis of 11 trials
Source: PLoS One. 2017 Jun 13;12(6):e0179335. doi: 10.1371/journal.pone.0179335 (PMC5469478; doi:10.1371/journal.pone.0179335)
Supplement: S2 Table — (DOCX) [file pone.0179335.s002.docx]

**S 2 Table.Quality assessment of these enrolled trials (see check list in Appendix 1)**

| **Author, year** | **Selection** | | | |  | **Comparability** | |  | **Outcome assessment** | | |
| --- | --- | --- | --- | --- | --- | --- | --- | --- | --- | --- | --- |
|  | **1** | **2** | **3** | **4** |  | **5** | **6** |  | **7** | **8** | **9** |
| Chung 2014 [13] | ☆ | ☆ | ☆ | - |  | ☆ | ☆ |  | ☆ | ☆ | - |
| Colak 2015 [14] | - | ☆ | ☆ | ☆ |  | ☆ | ☆ |  | - | ☆ | ☆ |
| Iribarren 2004 [17] | ☆ | ☆ | ☆ | - |  | ☆ | ☆ |  | - | ☆ | - |
| Iribarren 2012 [15] | ☆ | ☆ | ☆ | ☆ |  | ☆ | ☆ |  | ☆ | ☆ | - |
| Lee 2012 [12] | - | ☆ | ☆ | - |  | ☆ | ☆ |  | ☆ | ☆ | - |
| Liss 2000 [18] | - | - | - | - |  | ☆ | ☆ |  | - | ☆ | ☆ |
| Onufrak 2008 [19] | ☆ | ☆ | ☆ | ☆ |  | ☆ | ☆ |  | ☆ | ☆ | ☆ |
| Prosser 2010 [16] | - | - | ☆ | - |  | ☆ | ☆ |  | - | - | - |
| Schanen 2005 [20] | ☆ | ☆ | ☆ | ☆ |  | ☆ | ☆ |  | ☆ | ☆ | - |
| Toren 1996 [21] | ☆ | - | - | ☆ |  | - | - |  | ☆ | ☆ | ☆ |
| Yun 2012 [22] | - | - | - | - |  | ☆ | ☆ |  | - | ☆ | - |
