# Supplementary figures and images for: Association of asthma with coronary heart disease: A meta analysis of 11 trials
Source: PLoS One. 2017 Jun 13;12(6):e0179335. doi: 10.1371/journal.pone.0179335 (PMC5469478; doi:10.1371/journal.pone.0179335)

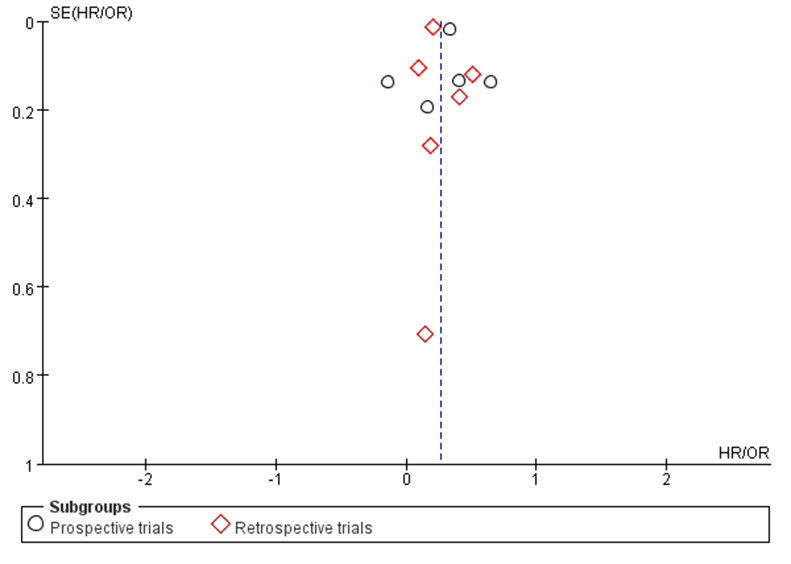

Supplement: S1 Fig — (TIF) [file pone.0179335.s003.tif]
